# Phenotypic and genotypic assessment of iron acquisition in diverse bovine-associated non-*aureus* staphylococcal strains

**DOI:** 10.1186/s13567-023-01260-z

**Published:** 2024-01-12

**Authors:** Helena Reydams, Bruno Toledo-Silva, Kristien Mertens, Sofie Piepers, Nick Vereecke, Fernando Nogueira Souza, Freddy Haesebrouck, Sarne De Vliegher

**Affiliations:** 1 M-Team and Mastitis and Milk Quality Research Unit, Department of Internal Medicine, Reproduction, and Population Medicine, Faculty of Veterinary Medicine, Ghent University, 9820 Merelbeke, Belgium; 2 Department of Translational Physiology, Infectiology and Public Health, Faculty of Veterinary Medicine, Ghent University, 9820 Merelbeke, Belgium; 3 PathoSense BV, Lier, Belgium; 4 Veterinary Clinical Immunology Research Group, Department of Internal Medicine, Faculty of Veterinary Medicine and Animal Sciences, University of São Paulo, Prof. Orlando Marques de Paiva Av. 87, São Paulo, 05508-270 Brazil; 5 Department of Pathobiology, Pharmacology and Zoological Medicine, Faculty of Veterinary Medicine, Ghent University, 9820 Merelbeke, Belgium

**Keywords:** Dairy cows, mastitis, non-*aureus* staphylococci, WGS, siderophore, iron-acquisition, lactoferrin, ferritin, *Staphylococcus chromogenes*, *Staphylococcus equorum*

## Abstract

Although the role of iron in bacterial infections has been well described for *Staphylococcus* (*S*.) *aureus*, iron acquisition in (bovine-associated) non-*aureus* staphylococci and mammaliicocci (NASM) remains insufficiently mapped. This study aimed at elucidating differences between four diverse bovine NASM field strains from two species, namely *S. chromogenes* and *S. equorum*, in regards to iron uptake (with ferritin and lactoferrin as an iron source) and siderophore production (staphyloferrin A and staphyloferrin B) by investigating the relationship between the genetic basis of iron acquisition through whole genome sequencing (WGS) with their observed phenotypic behavior. The four field strains were isolated in a previous study from composite cow milk (CCM) and bulk tank milk (BTM) in a Flemish dairy herd. Additionally, two well-studied *S. chromogenes* isolates originating from a persistent intramammary infection and from a teat apex were included for comparative purpose in all assays. Significant differences between species and strains were identified. In our phenotypical iron acquisition assay, while lactoferrin had no effect on growth recovery for all strains in iron deficient media, we found that ferritin served as an effective source for growth recovery in iron-deficient media for *S. chromogenes* CCM and BTM strains. This finding was further corroborated by analyzing potential ferritin iron acquisition genes using whole-genome sequencing data, which showed that all *S. chromogenes* strains contained hits for all three proposed ferritin reductive pathway genes. Furthermore, a qualitative assay indicated siderophore production by all strains, except for *S. equorum*. This lack of siderophore production in *S. equorum* was supported by a quantitative assay, which revealed significantly lower or negligible siderophore amounts compared to *S. aureus* and *S. chromogenes*. The WGS analysis showed that all tested strains, except for *S. equorum*, possessed complete staphyloferrin A (SA)-synthesis and export operons, which likely explains the phenotypic absence of siderophore production in *S. equorum* strains. While analyzing the staphyloferrin A and staphyloferrin B operon landscapes for all strains, we noticed some differences in the proteins responsible for iron acquisition between different species. However, within strains of the same species, the siderophore-related proteins remained conserved. Our findings contribute valuable insights into the genetic elements associated with bovine NASM pathogenesis.

**Supplementary Information:**

The online version contains supplementary material available at 10.1186/s13567-023-01260-z.

## Introduction

Bovine mastitis, an inflammation of the bovine mammary gland, is typically a result of bacterial intramammary infections (IMI), leading to important economic losses in dairy production worldwide [1]. Bovine-associated non-*aureus* staphylococci and the closely related mammaliicocci (NASM) [2] are traditionally considered to be commensals of the mammary gland or minor mastitis pathogens. Despite being the most prevalent group of bacteria cultured from aseptically collected milk samples of dairy cows, their role in the bovine mammary gland is under increasing scrutiny. Among and within bovine-associated NASM species, substantial variations have been observed on their effects on udder health and milk yield [3–5]. These include differences in virulence [6–8], potential beneficial properties [9, 10], host-interaction [11, 12], in vitro iron metabolism [8, 13], and epidemiological behavior [14, 15]. Additionally, differences have been reported regarding their ecology [15] culminating in the ecological categorization of specific NASM species as “host-adapted” (e.g., *Staphylococcus chromogenes*) or “environmental” (e.g., *Staphylococcus equorum*) [16]. However, there is also growing evidence of substantial strain-level variation within NASM species with some strains showing a greater propensity to adapt to specific hosts or even particular body sites [17].

Bacteria have a strict nutritional iron requirement for growth and pathogenesis (e.g., biofilm production). The concentration of free, bioavailable iron within the host, however, is restricted as a form of innate nutritional immunity against invading bacterial pathogens [18–20]. In response to iron-deplete conditions, staphylococci have developed multiple iron acquisition strategies from the extracellular environment including the secretion of siderophores. The latter are small (< 1 kDa) potent iron-chelating compounds with a high affinity for iron that compete with iron-binding host-derived glycoproteins such as lactoferrin (found in milk, mucosal secretions, and polymorphonuclear leucocytes) in the extracellular environment [21–23]. They are used by *Staphylococcus aureus*, a major mastitis pathogen, for iron acquisition [24–27]. Staphylococci can synthesize and secrete two hydroxycarboxylate type of siderophores, staphyloferrin A (SA) and staphyloferrin B (SB) encoded by a four-gene *sfaABCD* and nine-gene *sbnABCDEFGHI* operon, respectively [28]. Uptake of ferric-SA or ferric-SB is linked to non-interchangeable iron-regulated ABC-type transporters *htsABC* and *sirABC*, respectively, which are encoded by operons located near their respective siderophore biosynthetic genes [29, 30]. While the molecular basis of siderophore iron acquisition and its import into the cell has been studied extensively for *S. aureus* in vertebrate hosts [18, 20, 31], there is a paucity of information regarding siderophore production and the genetic basis of iron acquisition in (bovine-associated) NASM [32–35]. Also, as far as we know, only one report describes ferritin (an ubiquitous intracellular iron storage protein) as a potential staphylococcal iron acquisition mechanism (e.g., *S. xylosus* from meat) [36]. In this study, a three gene surface-associated reductase was implicated in iron ferritin acquisition rather than siderophore elaboration [36].

The competition for iron between a host and bacteria can determine the course and severity of the inflammatory reaction in response to the upcoming infection [23, 26]. In dairy cows, the concentration of lactoferrin in milk varies depending on udder health status, stage of lactation, and daily milk production [37]. An increase in milk ferritin concentrations during intramammary infection has been observed [38]. Still, bacterial iron scavenging in the mammary gland during an infection is not well understood [25]. Hence, elucidating iron acquisition mechanisms in bovine-associated NASM, including siderophore production and utilization of host-derived iron sources, will help better understand their role in udder health.

Substantial differences in iron acquisition have been observed between two different *S. chromogenes* strains: one originating from a persistent intramammary infection (the “IM” isolate) [39] and one from the teat apex of a dairy heifer (the “TA” isolate) [40]. The findings suggest *S. chromogenes* IM to be a true udder-adapted strain capable of acquiring iron to sustain growth in the mammary gland in contrast to *S. chromogenes* TA [13], and form the basis for further study of differences between and within other NASM species. Collectively, strain variation should be examined in complement to assessing the properties of different bovine-associated NASM to better understand their distribution across habitats and to elucidate their relevance for udder health and milk yield in dairy cows.

The aims of this study were (1) to investigate the capacity of two diverse *S. chromogenes* (ecologically classified as a “host-adapted” species) strains and two diverse *S. equorum* (ecologically classified as an “environmental” species) strains, originating from composite cow milk or bulk-tank milk, respectively, in utilizing different sources of iron; (2) to assess their siderophore production; and (3) to perform whole genome sequencing to identify their iron acquisition genes in descriptive comparison with their phenotypic behavior.

## Materials and methods

### Bacterial isolates

Four field NASM strains obtained from a previous study [41] and identified through matrix-assisted laser desorption/ionization time-of-flight mass spectrometry (MALDI-ToF MS) were included. The strains originated from composite cow milk (CCM) samples (one *S. chromogenes* CCM strain and one *S. equorum* CCM strain) and from bulk tank milk (BTM) samples (one *S. chromogenes* BTM strain and one *S. equorum* BTM strain), collected in tandem in one commercial dairy herd [41]. Isolates obtained in this previous study have been strain-typed by random amplification of polymorphic DNA polymerase chain reaction (RAPD-PCR). In the current study, the CCM and BTM strains from each species were selected based on having the lowest internal similarity scores (75.6% for isolates of both species), as calculated using the unweighted pair group method with arithmetic mean (UPGMA; Bionumerics software version 7.6.3).

Additionally, two well-studied *S. chromogenes* isolates originating from a persistent IMI lasting over 11 months (“IM”) [39] and from the teat apex of a dairy heifer (“TA”) [40] were included for comparative purpose in all assays as these two strains were previously used in iron assays in vitro and presented clear strain differences in multiple aspects [8, 9, 11, 13, 14, 40, 42, 43].

Quality control reference strain *S. aureus* ATCC 25923 [13, 26, 44] and *Escherichia coli* ATCC 25922 [45] served as positive controls for the phenotypical iron assay and the qualitative/quantitative siderophore production assay, respectively. *Streptococcus dysgalactiae* ATCC 43078 was used as a negative control for the qualitative siderophore production assay [26].

### Assays

#### Phenotypical iron test

The phenotypical iron test, in which the ability to acquire iron from host-binding proteins (ferritin and lactoferrin) is evaluated, was performed as described in Reydams et al. [13]. Briefly, the isolates were cultured overnight at 37 °C on Colombia blood agar with 5% sheep blood (CBA, Thermo Fisher Scientific). After reaching a density of 0.5 McFarland in separate 0.85% NaCl solution (Biomerieux), the bacterial cultures were diluted in Dulbecco's Phosphate Buffered Saline (Thermo Fisher Scientific) (dPBS). The isolates were subsequently grown in four different media including, trypticase soy broth (TSB) (Thermo Fisher Scientific), TSB deprived of iron by adding a final concentration of 0.5 mM of iron chelating agent 2–2’bipyridyl (dTSB) (Sigma Aldrich) [46], iron-deprived TSB supplemented with a final concentration of 50 μM ferritin from equine spleen (dTSBF) (Sigma Aldrich) [26, 36], and 0.4 mg/mL iron saturated recombinant human lactoferrin (dTSBL) (Sigma Aldrich) [13]. A 96-well microplate (Novolab) covered with a transparent seal was used for isolate growth in the different media for 24 h without agitation in a MultiSkan Go apparatus (Thermo Fisher Scientific). This allowed to measure the optical density (OD, 600 nm) 25 times with 1 h intervals to assess and quantify the bacterial growth. The incubation temperature was set at 37 °C for all isolates except for *S. equorum*, which showed optimal growth at 32 °C. Two replicates were taken for each isolate in the 96 well plate. The SkanIt 4.1 for Microplate Readers software (Thermo Fisher Scientific) was used for protocol input and recording of the results. The phenotypical iron-test was performed in duplicate on two independent days.

#### Qualitative siderophore production assay

The overlay (O) technique with chrome azurol S (CAS) medium (O-CAS) for siderophore detection was performed with some modifications [47]. In short, the blue CAS dye was prepared beforehand as described by Louden et al. [45] based on the original assay [48]. In preparation of O-CAS procedures, isolates were cultured for 24 h at 37 °C on CBA. After overnight incubation, pure colonies of each isolate were added to separate sterile 0.85% NaCl solution (Biomerieux) until a turbidity equivalent of 0.5 McFarland density was reached. With a sterile cotton swab, the inoculum was streaked on a quarter of two tryptic soy agar (TSA; Oxoid) plates. One of the plates was supplemented with 200 μM 2–2’ bipyridyl, while the other plate was not (serving as a negative control). Both plates were incubated overnight. Several concentrations of 2–2’bipyridyl were added to the TSA to determine optimal siderophore production without causing bacterial death. The medium for a liter of *overlay* was prepared according to Shin et al. [49]. In short, under stirring, 12 mL of 50% NaOH was added to 900 mL of ddH_2_O to dissolve 30.2 g of piperazine-N–N’bis(2-ethanesulfonic acid) (PIPES). After complete dissolution of PIPES in the *overlay*, 15 g of agarose (Sigma-Aldrich) was added and the *overlay* was autoclaved. Finally, 100 mL of the autoclaved blue CAS dye was mixed with the autoclaved *overlay* (under stirring) and applied over the TSA plates with(out) 200 μM 2–2’bipyridyl containing the cultivated isolates to be tested for siderophore production. After a minimum period of 15 min, a change in color (blue to yellow) was observed in the overlaid medium. This assay was performed twice on two independent days with two replicates (plates) for each isolate.

#### Quantitative siderophore production assay (modified microplate method)

Analysis of siderophore production were performed using the modified microplate method with some modifications [50]. Briefly, 50 μL of 0.5 McFarland of each isolate was placed in 5 mL of Iscove’s Modified Dulbecco’s Medium (IMDM, Thermo Fisher Scientific) and incubated at 37 °C for 48 h to induce maximal siderophore production. Afterwards, the supernatant was obtained by centrifugation at 10 000 rpm (12 298 × *g*) for 10 min. and filter sterilized (pore size 0.2 μm) (Puradisc Whatman FP30 CA-S, Avantor Life Sciences). Supernatant (100 μL) of each bacterial culture was added in separate wells of a 96 microplate followed by the addition of 20 μL of autoclaved CAS dye as described above. After a 20 min incubation period, the optical density of each sample was recorded at 660 nm using the Multiskan Go microplate reader. Three replicates were taken for each isolate in the 96 well plate and siderophore production, in percent siderophore unit (psu), was measured according to the following formula [50]:

$${\text{Siderophore}}\,{\text{production }}\left( {{\text{psu}}} \right){\text{ }} = \,\frac{{\left( {A_{r} - ~A_{s} } \right)~\,x\,~100}}{{A_{r} }}$$

where A_r_ = absorbance of reference (CAS solution with uninoculated broth), and A_s_ = absorbance of sample (CAS solution with cell-free supernatant of sample). The assay was performed four times in four independent days.

#### Whole genome sequencing, phylogenetic trees, and siderophore-related operon landscapes

The six NASM isolates (four field strains: *S. chromogenes* CCM, *S. chromogenes* BTM, *S. equorum* CCM, *S. equorum* BTM; two comparative strains: *S. chromogenes* IM, and *S. chromogenes* TA) were inoculated on CBA and delivered to the PathoSense laboratory at Ghent University for processing. The samples were processed to isolate High-Molecular Weight DNA as previously described [51–53]. Samples were multiplexed on an R9.4.1 flow cell (ONT) and sequenced using a GridION device [52]. Final genome assemblies were obtained using Trycycler (v.0.5.3) [54], minimap2 (v2.20) [55], and medaka (v.1.7.3; ONT) as described already for staphylococci before [56]. Resulting bacterial genome assemblies were used in a single nucleotide polymorphism (SNP)-based phylogenetic inference using csi phylogeny [57] and IQtree (v.1.6.12) [58, 59] with –bb 1000 and -m GTR + R + I settings. The Bioproject for this study is PRJNA1008278 and the associated NCBI accession numbers are: CP133240-CP133241 (*S. chromogenes* CCM), CP133242-CP133243 (*S. chromogenes* BTM), CP133235-CP133239 (*S. equorum* CCM), CP133229-CP133234 (*S. equorum* BTM), CP133244-CP133246 (*S. chromogenes* IM), and CP133247-CP133248 (*S. chromogenes* TA).

This analysis was supplemented with complete *S. chromogenes* (n = 89) and *S. equorum* (n = 60) genome sequences as available from Naushad et al. [35], a Canadian database. Also, the *S. aureus* ATCC 25923 (CP009361) was included for comparison. All genomes were screened for siderophore and iron-uptake associated genes using a custom protein database adapted from Naushad et al. [35] and Vermassen et al. [36] (see Additional file 1) in Abricate (v.1.0.1) [69] with minimal query coverage and amino acid homology set to 30 and 50%, respectively. The protein sequences for siderophore-related and ferritin iron acquisition were obtained from Naushad et al. [35] and Vermassen et al. [36] with *S. aureus* and *S. xylosus* as reference, respectively. The first genomic hits that met the minimum cutoff for each individual query were selected. Trees and identified proteins were visualized in iTOL (v.5) [60]. To study the siderophore-related operon landscape, flanking regions (20 000 bp up- and downstream) of target genes were extracted using flanker (v.0.1.5) [61]. Subsequent sequences were annotated with Bakta (v.1.7.0) [62] and visualized with Clinker (v.0.0.26) [63].

### Statistical analysis

#### Phenotypical iron test

The growth of the four field strains (*S. chromogenes* CCM, *S. chromogenes* BTM, *S. equorum* CCM, and *S. equorum* BTM), the comparative strains (*S. chromogenes* IM, *S. chromogenes* TA), and the positive control *S. aureus* ATCC25923, in the different growth media was expressed as the area under the curve (AUC) [13]. The association between the AUC (outcome variable) and the different growth media (categorical predictor variable: TSB, dTSB, dTSBF, and dTSBL) and the bacterial strains (categorical predictor variable) was determined fitting a linear mixed regression model (PROC MIXED, SAS version 9.4, SAS Institute Inc., Cary, NC, USA). The interaction term between the growth media and strains was tested and isolate was added as random effect to account for the correlation among the duplicates in the experiment.

#### Quantitative siderophore production assay

The expression of siderophores (psu; outcome variable) during incubation with different bacterial strains (the four field strains *S. chromogenes* CCM, *S. chromogenes* BTM, *S. equorum* CCM, *S. equorum* BTM and the two comparative strains: *S. chromogenes* IM, *S. chromogenes* TA; and the positive controls *S. aureus* ATCC 25923 and *E. coli* ATCC 25922; categorical predictor variable of main interest) was studied by fitting a linear regression model (PROC MIXED, SAS version 9.4) considering the triplicates and rounds as fixed effects.

The significance level was set at *P* ≤ 0.05 for both analyses. In all analyses, a Bonferroni correction was applied to adjust for multiple comparisons.

## Results

### Phenotypical iron assay

**Figure 1:**
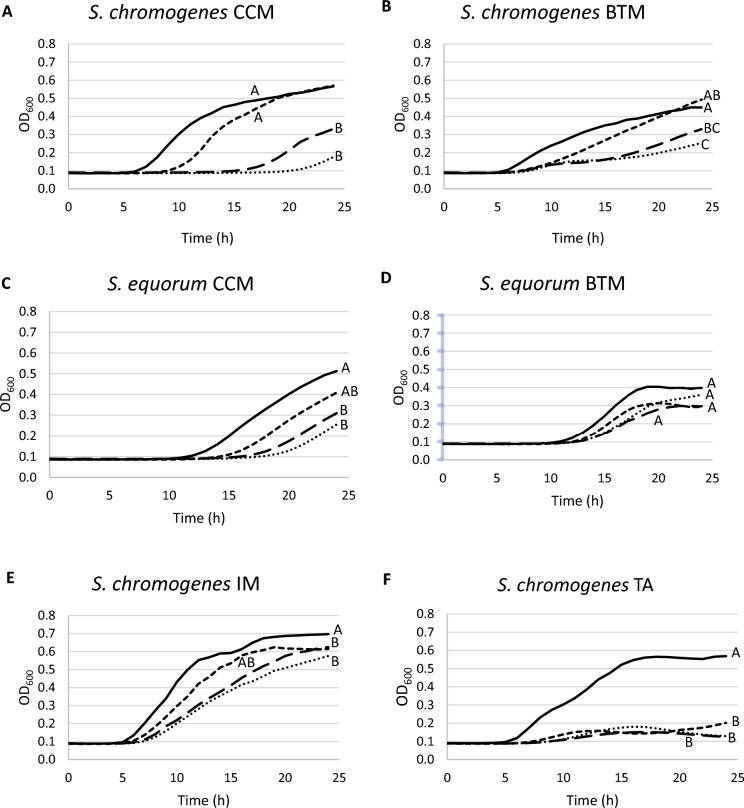
**Overview of strain growth (optical density, OD600) over 24 h in different growth media.** The four field strains, *Staphylococcus chromogenes* from composite cow milk (CCM) and from bulk tank milk (BTM) (**A**, **B**) and *S. equorum* from CCM and BTM **(C**, **D**), and the two comparative strains, *Staphylococcus chromogenes* isolates from a persistent intramammary infection (IM) and from the teat apex of a dairy heifer (TA) (**E**, **F**), are grown in tryptic soy broth (TSB, solid line), deferrated tryptic soy broth (dTSB, dotted line), deferrated tryptic soy broth with ferritin from equine spleen (dTSBF, short-dash line), and deferrated tryptic soy broth with human recombinant lactoferrin (dTSBL, long-dash line). All experiments were performed in duplicate. Different letters within each figure (**A**–**C**) indicate significant differences when applying the Bonferroni correction between growth media within strains (*P* ≤ 0.05).

Bacterial growth differed significantly between strains (*P* < 0.001) and media (*P* < 0.001) (Table 1). Overall, both *S. chromogenes* field strains, CCM and BTM, presented a better growth across all media [Least-square means (LSM) of the AUC = 5.00 and 4.79, respectively] when compared to the *S. equorum* strains CCM and BTM (LSM = 3.51 and 4.15, respectively). When comparing the field strains with the comparative NASM strains, *S. chromogenes* IM presented the highest growth across all media that was not significantly different from the positive control *S. aureus* ATCC 25923 strain (LSM = 8.55; *P* = 0.92). On the other hand, *S. chromogenes* TA, presented lower growth than *S. chromogenes* CCM and BTM, while overall higher growth than both *S. equorum* strains (LSM = 4.47) was observed. Strains grew the best on TSB (LSM = 7.45; Table 1). Media with ferritin as an iron source (LSM = 5.71) resulted in significantly better growth than dTSB (LSM = 4.35; Bonferroni-corrected *P* < 0.001) and dTSBL (LSM = 4.80; Bonferroni-corrected *P* < 0.001) across all species. Still, growth of the strains was influenced by the type of media (interaction term between media and strains: *P* < 0.001; Table 1, Figure 1, and Additional file 2) with both *S. chromogenes* field strains, CCM and BTM, presenting significantly improved growth recovery with ferritin as an added iron source (Bonferroni-corrected *P* < 0.001 and Bonferroni-corrected *P* = 0.003, respectively), but not with lactoferrin (Bonferroni-corrected *P* = 1.000, both) (Figures 1A, B). Growth of both *S. equorum* field strains (Figures 1C, D) are not significantly influenced by the different media, except for *S. equorum* CCM when comparing growth in TSB to dTSB (Bonferroni-corrected *P* = 0.0002) (Figure 1C). Regarding the comparative strains, both *S. chromogenes* IM and TA showed significant reduction in maximum growth in dTSB (Bonferroni-corrected *P* < 0.001, both) when compared to growth in TSB and growth was not significantly recovered when ferritin (Bonferroni-corrected *P* = 0.49 and *P* = 1.000, respectively) or lactoferrin (Bonferroni-corrected *P* = 1.000, both) was added as an iron source (Figures 1E, F).

**Table 1:** **Linear mixed regression model for the area under the curve (AUC) of the phenotypical iron assay**

	β^a^	SE^b^	LSM^c^	*P*^d^
Intercept	9.23	0.42		< 0.001^e^
Medium				< 0.001^d^
TSB	Ref.^e^	–	7.45	–
dTSB	−1.21	0.57	4.35	< 0.001^e^
dTSBF	− 1.13	0.57	5.71	< 0.001^e^
dTSBL	0.14	0.57	4.80	< 0.001^e^
Strain				< 0.001^d^
* S. aureus* ATCC 25923	Ref.	–	8.58	–
* S. chromogenes* IM^f^	1.27	0.57	8.55	0.92^e^
* S. chromogenes* TA^g^	−0.61	0.57	4.47	< 0.001^e^
* S. chromogenes* from CCM^h^	−1.23	0.49	5.00	< 0.001^e^
* S. chromogenes* from BTM^i^	−2.75	0.49	4.79	< 0.001^e^
* S. equorum* from CCM^j^	−4.25	0.49	3.51	< 0.001^e^
* S. equorum* from BTM^k^	−4.17	0.49	4.15	< 0.001^e^
Medium * Strain^l^				< 0.001^d^

^a^Regression coefficient.

^b^Standard error.

^c^Least square means.

^d^Overall *P*-value for fixed effect.

^e^*P*-value for differences of least square means.

^f^*Staphylococcus chromogenes* isolate causing chronic intramammary infection [39].

^g^*Staphylococcus chromogenes* isolate from a teat apex of a heifer [40].

^h^*Staphylococcus chromogones* isolate from composite cow milk [41].

^I^*Staphylococcus chromogones* isolate from bulk tank milk [41].

^j^*Staphylococcus equorum* isolate from composite cow milk [41].

^k^*Staphylococcus equorum* isolate from bulk tank milk [41].

^l^Interaction medium * strain; see Figure 1.

### Qualitative siderophore production assay

*Staphylococcus aureus* ATCC 25923, *S. chromogenes* IM, *S. chromogenes* TA, and both *S. chromogenes* isolates from CCM and BTM exhibited yellow coloration indicative for the production of iron chelators. For both *S. equorum* isolates, no iron chelator activity was observed.

### Quantitative siderophore production assay

There was a significant strain effect (*P* < 0.001; Table 2) with *S. aureus* ATCC 25923 producing significantly higher amounts of siderophores [Least-square means (LSM) of the psu = 74.5] when compared to all NASM strains (Bonferroni corrected *P* < 0.0001). The difference in psu between *S. aureus* ATCC 25923 and positive control *E. coli* 25922 borders on significance (LSM = 62.5; *P* = 0.06). Overall, the *S. chromogenes* strains from CCM and BTM produced a higher amount of siderophores (LSM = 29.8 and 14.7, respectively) when compared to the *S. equorum* strains from CCM and BTM (LSM = 3.66 and 3.11, respectively). *Staphylococcus chromogenes* from CCM presented a higher siderophore production when compared to both *S. equorum* strains (Bonferroni corrected *P* = 0.007 and Bonferroni corrected *P* = 0.005 from CCM and BTM, respectively; see Additional file 3) and *S. chromogenes* TA (Bonferroni corrected *P* = 0.004). Both *S. chromogenes* (Bonferroni corrected *P* = 0.58) and *S. equorum* (Bonferroni corrected *P* = 1.000) from CCM had a higher psu when compared to strains of the same species from BTM although differences between the two strains of the same species were insignificant. *Staphylococcus chromogenes* IM siderophore production (LSM = 21.9; Bonferroni corrected *P* = 0.09) was not significantly different from *S. chromogenes* TA (LSM = 2.1).

**Table 2:** **Linear mixed regression model for the percentage siderophore units (psu) of the quantitative microplate analysis**

	Quantitative analysis			
	β^a^	SE^b^	LSM^c^	*P*^d^
Intercept	62.0	4.37		< 0.001^d^
Strain				< 0.001^d^
* E. coli* ATCC 25922	Ref.	–	62.52	–
* S. aureus* ATCC 25923	12.02	6.07	74.53	0.0594^e^
* S. chromogenes* CCM^f^	−32.71	6.07	29.80	< 0.001^e^
* S. chromogenes* BTM^g^	−47.73	6.07	14.79	< 0.001^e^
* S. equorum* CCM^h^	−58.85	6.07	3.66	< 0.001^e^
* S. equorum* BTM^i^	−59.40	6.07	3.12	< 0.001^e^
* S. chromogenes* IM^j^	−40.65	6.07	21.86	< 0.001^e^
* S. chromogenes* TA^k^	−60.41	6.07	2.10	< 0.001^e^

^a^Regression coefficient.

^b^Standard error.

^c^Least square means.

^d^Overall *P*-value for fixed effect.

^e^*P*-value for differences of least square means.

^f^*Staphylococcus chromogenes* isolate from composite cow milk [41].

^g^*Staphylococcus chromogenes* isolate from bulk tank milk [41].

^h^*Staphylococcus equorum* isolate from composite cow milk [41].

^i^*Staphylococcus equorum* isolate from bulk tank milk [41].

^j^*Staphylococcus chromogenes* isolate causing chronic intramammary infection [39].

^k^*Staphylococcus chromogenes* isolate from a teat apex of a heifer [40].

### Whole-genome sequencing

#### Phylogenetic analysis

**Figure 2:**
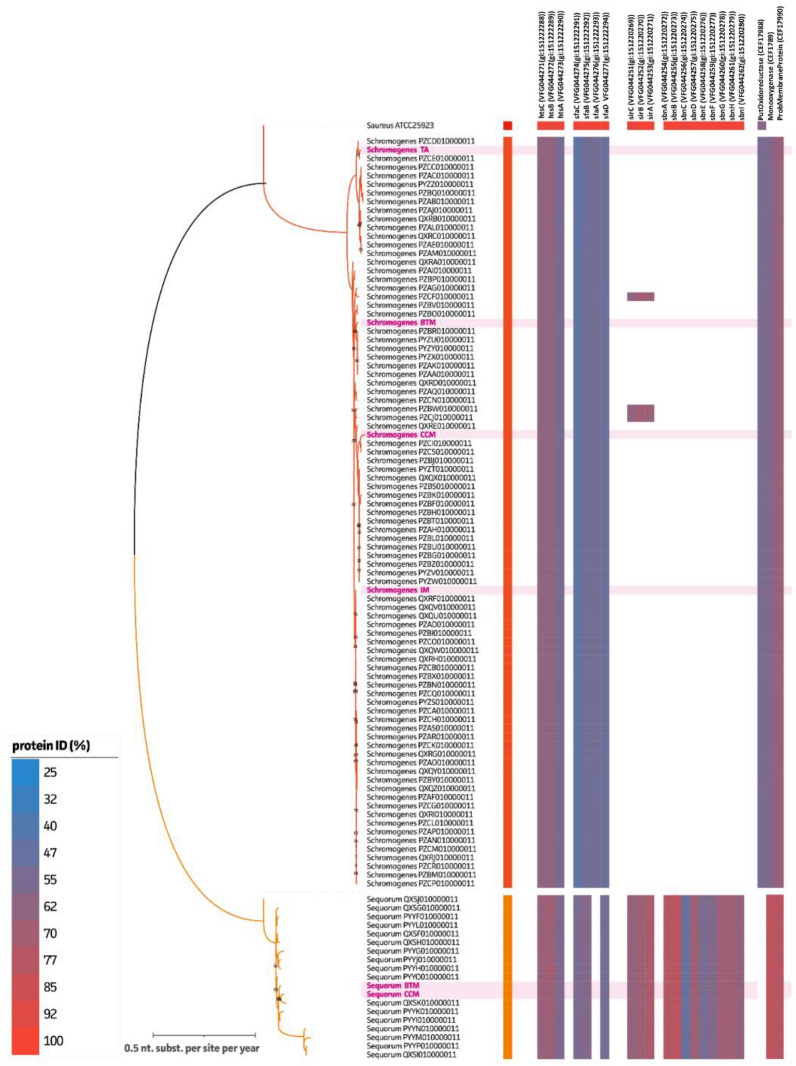
**Siderophore-associated protein homology in diverse NASM isolates.** Presence of siderophore-associated proteins within *S. chromogenes* isolates [from a persistent intramammary infection (IM), the teat apex of a dairy heifer (TA), composite cow milk (CCM) and bulk tank milk (BTM)] and *S. equorum* isolates (from CCM and BTM) highlighted in pink, including 100 isolates (*S. chromogenes* = 83*, S. equorum* = 17) from the Mastitis Pathogen Collection of the Canadian Bovine Mastitis and Milk Quality Research Network (CBMQRN) [35], and quality control reference strain *S. aureus* ATCC 25923. An ML tree (1000 ultrafast bootstraps) representing phylogenetic relationship of *S. chromogenes*, *S. equorum*, and quality control strain *S. aureus* ATCC 25923 on whole genome SNP level with the *S. aureus* ATCC 25923 (CP009361) as reference. The final phylogenetic tree was annotated with the presence of siderophore- and ferritin-related protein hits across genomes. Color coded (blue-red) represents amino acid homology of the identified proteins as compared to the NCBI siderophore- and ferritin-related protein hits. Only hits with amino acid homology above 30% and 50% query coverage are shown.

The SNP-based WGS phylogenetic analysis (Figure 2; Additional file 4) including our field strains (*S. chromogenes* CCM, *S. chromogenes* BTM, *S. equorum* CCM, and *S. equorum* BTM), our comparative strains (*S. chromogenes* IM and *S. chromogenes* TA), and the Canadian *S. chromogenes* and S. equorum isolates [40] divided our four field isolates and two comparative isolates into two distinct clades: *S. chromogenes* TA in a separate clade from the other three *S. chromogenes* isolates (IM, CCM, and BTM] and the two *S. equorum* isolates (CCM and BTM] in one clade but still divergent.

#### Identification of siderophore-associated genes and operon landscapes

**Figure 3:**
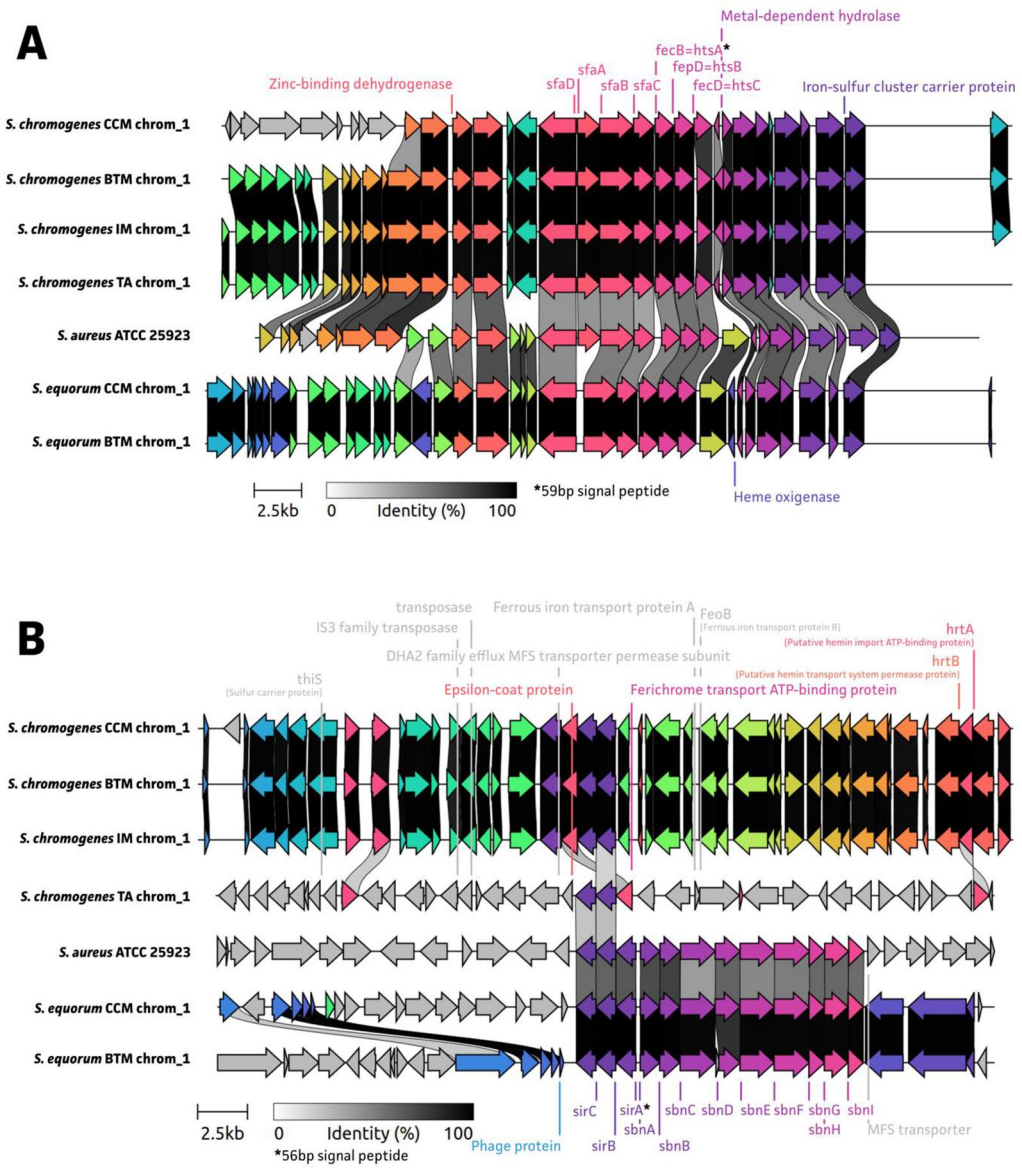
**Siderophore-related** ***sfa-hts* (A) and *sbn-sir*(B) operon landscapes.** Operon landscapes for the four field strains, *Staphylococcus chromogenes* from composite cow milk (CCM) and from bulk tank milk (BTM) and *S. equorum* from CCM and BTM, the two comparative strains, *S. chromogenes* isolated from a persistent intramammary infection (IM) and from the teat apex of a dairy heifer (TA), and positive control, *Staphylococcus aureus* ATCC 25923.

Based on the WGS data of the four field strains and two comparative strains (Figures 2, 3), it was observed that all *S. chromogenes* and *S. equorum* strains, including the reference strain *S. aureus* ATCC 25923, contained all SA receptor *hts* operon proteins (htsABC). Both *S. equorum* isolates did not have a complete SA synthesis related *sfa* operon (sfaABCD) embedded in their genomes in contrast to the four *S. chromogenes* isolates (and reference strain *S. aureus* ATCC 25923). Similar to *S. aureus* ATCC 25923, both *S. equorum* isolates showed the presence of both SB receptor sir operon proteins (sirABC) and SB synthesis-related sbn operon proteins (sbnABCDEFGHI) in contrast to all the *S. chromogenes* isolates.

Regarding the potential ferritin reductive pathway for iron acquisition, when using *S. xylosus* as reference strain, the four *S. chromogenes* isolates had protein hits for the three genes encoding proteins potentially contributing to ferritin iron acquisition: a putative oxidoreductase protein, mono-oxygenase protein, and probable membrane protein showing a mean of 55.4% (standard deviation, SD ± 0.15%), 58.7% (± 0.31%), and 66.6% (± 0.00%) amino acid homology, respectively. Both *S. equorum* isolates did not have a hit for the putative oxidoreductase while both other hits (mono-oxygenase protein and probable membrane protein) showed higher protein homologies when compared to the *S. chromogenes* isolates (mono-oxygenase protein and probable membrane protein showing a mean of 79.8% (± 0.00%) and 77.62% (± 0.00%) AA homology, respectively). In the *S. aureus* ATCC 25923 genome, only one protein hit (putative oxidoreductase) was identified (60.36% AA homology).

When taking a closer look at the *sfa-hts* (Figure 3A) and *sbn-sir* (Figure 3B) operon landscape, we observed a lower protein homology between species (~50%) but near 100% protein match between strains of the same species. Interestingly, a zinc-binding hydrogenase, metal-dependent hydrolase, and iron-sulfur cluster carrier protein were identified in the vicinity of the hts-sfa operons. Ferrous iron transport-related proteins (Feo A and FeoB) [64] were identified in *S. chromogenes* TA (Figure 3B) and in the *S. equorum* genomes also a heme oxygenase was identified (Figure 3A). Even though the sir-sbn operons were only complete in *S. aureus* and *S. equorum* strains, the four *S. chromogenes* genomes also showed protein homologs of both sirB and sirC. The latter proteins showed again the presence of various iron-associated proteins, including hrtA, hrtB, and ferichrome transport ATP-binding protein (Figure 3B).

## Discussion

Our study integrated the phenotype and genotype of different bovine-associated NASM strains when assessing their iron metabolism. By doing so, we observed substantial differences between species and strains. In the phenotypical iron assay, ferritin was an effective iron source for growth recovery in iron-deficient media for the *S. chromogenes* CCM and BTM strains. This finding was further supported by the examination of potential ferritin iron acquisition genes based on WGS data, as all *S. chromogenes* strains displayed hits for all three proposed ferritin reductive pathway genes. For the qualitative siderophore production assay, a color change was observed in all strains except for *S. equorum*, suggesting the latter species does not produce siderophores. This observation is further supported by the quantitative assay, in which this species produced little or negligible amounts of siderophores when compared to *S. aureus* and *S. chromogenes*. The WGS analysis revealed that all tested strains, except for *S. equorum*, possess complete SA-synthesis and export *sfa* operons, which could explain the phenotypic absence of siderophore production in both *S. equorum* strains. When analyzing the *sfa-hts* and *sbn-sir* operon landscapes for all strains, some interspecific variation in protein identities responsible for iron acquisition were observed but between strains of the same species the siderophore-related proteins are conserved. The results contribute to the currently limited understanding of the genetic elements associated with bovine NASM pathogenesis.

Importantly, the iron-metabolism related virulence genes profiles of our six NASM isolates (four field strains and two comparative strains) coincides with the virulence gene profiles of *S. chromogenes* and *S. equorum* isolates from a vast collection of Canadian [65] bovine isolates based on comprehensive WGS data. It must however, be taken into consideration that the protein sequences for iron acquisition reported in this study were based on specific criteria described in a previous study (i.e., applying a minimum of 30% amino acid identity and 50% query coverage) [35]. This means that the results obtained from these isolates may not be the same as when applying a higher cut-off for protein sequence homology as done in other studies [53, 56, 66]. Nevertheless, our findings combined with in vitro phenotypical iron assays offer novel insights into iron acquisition of ecologically different bovine-associated NASM species.

When assessing the growth curves of the four field strains, *S. chromogenes* CCM, *S. chromogenes* BTM, *S. equorum* CCM, and *S. equorum* BTM, and the two comparative strains, *S. chromogenes* IM and TA, in the four different growth media, all strains appear to have distinct phenotypic growth patterns. This observation is supported by the reported significant strain effect on bacterial growth and aligns with a previous study initially reporting significant strain differences between *S. chromogenes* IM and TA in the phenotypical iron assay [13]. Interestingly, both *S. chromogenes* strains from CCM and BTM demonstrated a significant ability to utilize ferritin for growth recovery, which was not observed in the *S. equorum* strains and the two comparative *S. chromogenes* strains. Although the initial differences in growth in different media may seem negligible, we observed phenotypic variations in the lag phase of *S. equorum* from CCM, suggesting a potential adaptation to utilizing iron-bound proteins to support growth. On the other hand, comparative strain *S. chromogenes* IM did not show significant growth recovery with iron supplementation under iron-deprived conditions this time, indicating that this specific strain might be less adaptable to iron-deprived circumstances than previously hypothesized [13]. However, it is worth noting that the AUC for *S. chromogenes* IM in dTSB media was higher than in the aforementioned study, suggesting that our strain was indeed able to adapt to iron-deprived media to a certain degree, possibly by sufficiently maintaining intracellular iron levels for proliferation [36]. As for *S. chromogenes* TA, our findings once again demonstrated the strain’s inability to exploit multiple iron sources.

It is generally accepted that the siderophore SA synthesis and transport related operons are found in most staphylococcal genomes, while the siderophore SB synthesis and transport related operons are predominantly found in the genomes of *S. aureus* [18]. The presence of the *sfa* operon was confirmed for all *S. chromogenes* and *S. equorum* isolates; however our *S. equorum* isolates appear to contain a deletion of the *sfa*A gene, an efflux transporter responsible for the export of SA into the extracellular milieu [67]. Interestingly, in contrast to the *S. chromogenes* strains, for all *S. equorum* strains, a complete 9-gene-*sfa*-operon was identified. When examining siderophore production, phenotypically *S. aureus* ATCC 25923 and all *S. chromogenes* isolates were positive for siderophore production in the qualitative assay with *S. aureus* producing the highest amount of siderophores followed by the *S. chromogenes* isolates in the quantitative assay. This was expected for *S. aureus* as they are known siderophore producers with SB the most robustly upregulated within the iron-restricted host [18]. In our study, 100% AA hits was found for all studied iron acquisition genes in the genotype of the quality control strain *S. aureus* ATCC 25923 when compared to the *S. aureus* reference strain used in WGS. *Staphylococcus chromogenes* in general presented quantitatively a lower and higher siderophore production than *S. aureus* and *S. equorum*, respectively. This was expected, because the *S. chromogenes* strains are considered to be “host-adapted” in an ecological context and carry all SA-related genes, including the *sfa*A gene for SA export. It is speculated that SA synthesis has a limited ability to transport iron into the cells [21] and in contrast to SB, its production is severely hampered in iron-limited, glucose-containing media [18]. Serum has a lower iron and higher glucose content when compared to, for example, the skin, which would elucidate the lower siderophore production of our NASM strains when compared to *S. aureus* ATCC25923 and support the general consensus of NASM species as commensals rather than invasive. Interestingly, both *S. chromogenes* isolates from IM and CCM appear to have a higher siderophore production when compared to the isolates from TA and BTM. Even though these differences were statistically not significant, when taking a closer look at the *sfa-hts* operon landscape, we again see variation in genes around the siderophore-associated genes for *S. chromogenes* TA when compared to the other *S. chromogenes* strains from IM, CCM, and BTM. This variation could be part of the reason this strain has presented different characteristics when compared with the IM strain in multiple assays [8, 9, 11, 13, 14, 40, 42, 43]. Notably, the *S. equorum* strains had hits for all SB genes and consequently, one would anticipate that the strains within this species would be strong siderophore producers similar to *S. aureus* ATCC 25923. However, based on our findings, *S. equorum* isolates did not exhibit high production of siderophores. It is conceivable that the in vitro assay might lack the required sensitivity to detect SB synthesis effectively or that the *sbn*-*sir* operon is not being expressed under the current experimental conditions. Additionally, it is also plausible that these proteins related to SB production have undergone functional divergence in these strains. The latter however, seems unlikely because although we can see an evolutionary change based on the phylogenetic hits (% AA identity), the *sfa-hts* and *sbn-sir* operons are likely to share significant functional similarities. When considering the proteins adjacent to the *sbn-sir* operon protein, there is significant variation in genes present between *S. aureus* ATCC 25923 and both *S. equorum* isolates which could explain our observed lack of siderophore production in *S. equorum*.

Regarding ferritin iron acquisition, we observed significant growth recovery for both *S. chromogenes* field strains and visually, a shortened lag phase for *S. equorum* CCM, which would suggest that a mechanism is present to access ferritin iron. The phylogenetic protein hits for the proposed model responsible for ferritin iron acquisition, would appear to support the phenotypes we observed. Although we did not observe complete hits for *S. equorum* and *S. aureus* ATCC 25923, our findings do not necessarily exclude the capability of these isolates to acquire ferritin iron. Either the reference strain was not sufficient to make comparisons or other mechanisms for ferritin iron acquisition might be employed.

Performing SNP-based phylogenetic inference and supplementing the current data with WGS data of bovine-associated *S. chromogenes* and *S. equorum* isolates from a Canadian study provided new insights in the genetic diversity and strain-relatedness of bovine-associated *S. chromogenes* and *S. equorum*. The *S. chromogenes* isolates, specifically IM and TA were, in line with previous studies applying MLST strain typing schemes [68], confirmed to be two unique strains, as well as belonging to two distinct genomic clades when including the collection of Canadian bovine *S. chromogenes* isolates. The two *S. chromogenes* isolates from CCM and BTM were found to belong to the same clade as the *S. chromogenes* IM isolate. Similar to a previous study that utilized RAPD strain typing [41], it was confirmed that these isolates are distinct strains with a significant distance in the phylogenetic tree between them. For the *S. equorum* isolates, WGS confirmed the isolates to be of two different strain types previously determined with RAPD-PCR [41] albeit with a closer relationship in the phylogenetic tree than the *S. chromogenes* isolates.

Collectively, our study emphasizes the importance of complementing the analysis of putative virulence factor genes with phenotypic testing. While our findings mainly highlight differences at the species level, we observed distinct interstrain growth and we still believe it is essential to consider strain-level variation within species when assessing NASM. Although our sample size was limited, we believe our findings provide a groundwork for future research on the importance of NASM for udder health and by extension, animal and public health. Furthermore, it is crucial to understand the mechanism of iron acquisition in NASM and the genetic basis underlying it. There are other iron acquisition mechanisms worth exploring, such as the uptake of heme–iron from hemoglobin, an important iron source for *S. aureus* and contributes significantly to pathogenesis (34). Our preliminary data on heme–iron acquisition genes (data not shown) for the strains in our study suggests that this mechanism is unlikely to be utilized by them. However, a different NASM species, (human-associated) *Staphylococcus lugdunensis*, was recently discovered having a functional heme–iron uptake system (34). These findings underscores the variability in iron acquisition strategies among different staphylococcal species and could have implications for their ecological niches and pathogenic potential. This knowledge is essential for understanding the relevance of these organisms to udder health and for addressing the growing threat of antimicrobial resistance, as these pathways may provide an alternative avenue for therapeutic approaches in treating mastitis.

### Supplementary Information

**Additional file 1: Sequences of virulence factors data set of staphylocococcal siderophore- and ferritin-related iron acquisition.**

Additional file 2: Multiple comparisons for the interaction term of the statistical analysis for medium * strain. This includes four field strains: S. chromogenes CCM (SCH CCM), S. chromogenes BTM (SCH BTM), S. equorum CCM (SEQ CCM), and S. equorum BTM (SEQ BTM); two comparative strains: S. chromogenes IM (SCH IM) and S. chromogenes TA (SCH TA); one positive control: Staphylococcus aureus ATCC 25923 (SA). All isolates are grown in 4 different types of media: an iron-rich medium namely trypticase soy broth (TSB), TSB deprived of iron by adding an iron chelating agent 2-2’bipyridyl (dTSB), iron-deprived TSB supplemented with ferritin derived from equine spleen (dTSBF) and iron saturated recombinant human lactoferrin (dTSBL).

**Additional file 3: Multiple comparisons for the strains from the statistical analysis for strain effect.** This includes four field strains: S. chromogenes CCM (SCH CCM), S. chromogenes BTM (SCH BTM), S. equorum CCM (SEQ CCM), and S. equorum BTM (SEQ BTM); two comparative strains: S. chromogenes IM (SCH IM) and S. chromogenes TA (SCH TA); two positive controls: Escherichia coli ATCC 25922 (EC) and Staphylococcus aureus ATCC 25923 (SA).

**Additional file 4: Comparative genomic analysis of the four field strains and two comparative strains, an overview.**
