# Supplementary material for: Phenotypic and genotypic assessment of iron acquisition in diverse bovine-associated non-aureus staphylococcal strains
Source: Vet Res. 2024 Jan 12;55:6. doi: 10.1186/s13567-023-01260-z (PMC10785429; doi:10.1186/s13567-023-01260-z)
Supplement: Supplementary file 2 — Additional file 2: Multiple comparisons for the interaction term of the statistical analysis for medium * strain. This includes four field strains: S. chromogenes CCM (SCH CCM), S. chromogenes BTM (SCH BTM), S. equorum CCM (SEQ CCM), and S. equorum BTM (SEQ BTM); two comparative strains: S. chromogenes IM (SCH IM) and S. chromogenes TA (SCH TA); one positive control: Staphylococcus aureus ATCC 25923 (SA). All isolates are grown in 4 different types of media: an iron-rich medium namely trypticase soy broth (TSB), TSB deprived of iron by adding an iron chelating agent 2-2’bipyridyl (dTSB), iron-deprived TSB supplemented with ferritin derived from equine spleen (dTSBF) and iron saturated recombinant human lactoferrin (dTSBL). [file 13567_2023_1260_MOESM2_ESM.docx]

| **Strain** | **Medium** | **Strain** | **Medium** | **β^a^** | **SE^b^** | **Adj P^c^** |
| --- | --- | --- | --- | --- | --- | --- |
| SCH BTM**^d^** | dTSB | SCH BTM | dTSBF | -1.9680 | 0.4011 | 0.0030 |
| SCH BTM | dTSB | SCH BTM | dTSBL | -0.3703 | 0.4011 | 1.0000 |
| SCH BTM | dTSB | SCH BTM | TSB | -2.9125 | 0.4011 | <.0001 |
| SCH BTM | dTSBF | SCH BTM | dTSBL | 1.5978 | 0.4011 | 0.0727 |
| SCH BTM | dTSBF | SCH BTM | TSB | -0.9445 | 0.4011 | 1.0000 |
| SCH BTM | dTSBL | SCH BTM | TSB | -2.5423 | 0.4011 | <.0001 |
| SCH CCM**^e^** | dTSB | SCH CCM | dTSBF | -4.3410 | 0.4011 | <.0001 |
| SCH CCM | dTSB | SCH CCM | dTSBL | -0.9021 | 0.4011 | 1.0000 |
| SCH CCM | dTSB | SCH CCM | TSB | -5.6229 | 0.4011 | <.0001 |
| SCH CCM | dTSBF | SCH CCM | dTSBL | 3.4389 | 0.4011 | <.0001 |
| SCH CCM | dTSBF | SCH CCM | TSB | -1.2819 | 0.4011 | 0.8539 |
| SCH CCM | dTSBL | SCH CCM | TSB | -4.7208 | 0.4011 | <.0001 |
| SEQ BTM | dTSB | SEQ BTM | dTSBF | -0.05925 | 0.4011 | 1.0000 |
| SEQ BTM**^f^** | dTSB | SEQ BTM | dTSBL | 0.2589 | 0.4011 | 1.0000 |
| SEQ BTM | dTSB | SEQ BTM | TSB | -1.0188 | 0.4011 | 1.0000 |
| SEQ BTM | dTSBF | SEQ BTM | dTSBL | 0.3181 | 0.4011 | 1.0000 |
| SEQ BTM | dTSBF | SEQ BTM | TSB | -0.9595 | 0.4011 | 1.0000 |
| SEQ BTM | dTSBL | SEQ BTM | TSB | -1.2776 | 0.4011 | 0.8809 |
| SEQ CCM**^g^** | dTSB | SEQ CCM | dTSBF | -1.0237 | 0.4011 | 1.0000 |
| SEQ CCM | dTSB | SEQ CCM | dTSBL | -0.3480 | 0.4011 | 1.0000 |
| SEQ CCM | dTSB | SEQ CCM | TSB | -2.2831 | 0.4011 | 0.0002 |
| SEQ CCM | dTSBF | SEQ CCM | dTSBL | 0.6757 | 0.4011 | 1.0000 |
| SEQ CCM | dTSBF | SEQ CCM | TSB | -1.2594 | 0.4011 | 1.0000 |
| SEQ CCM | dTSBL | SEQ CCM | TSB | -1.9351 | 0.4011 | 0.0040 |
| SCH IM**^h^** | dTSB | SCH IM | dTSBF | -1.9165 | 0.5673 | 0.4947 |
| SCH IM | dTSB | SCH IM | dTSBL | -0.6670 | 0.5673 | 1.0000 |
| SCH IM | dTSB | SCH IM | TSB | -3.3297 | 0.5714 | <.0001 |
| SCH IM | dTSBF | SCH IM | dTSBL | 1.2495 | 0.5673 | 1.0000 |
| SCH IM | dTSBF | SCH IM | TSB | -1.4132 | 0.5714 | 1.0000 |
| SCH IM | dTSBL | SCH IM | TSB | -2.6627 | 0.5714 | 0.0072 |
| SCH TA**^i^** | dTSB | SCH TA | dTSBF | -0.1183 | 0.5673 | 1.0000 |
| SCH TA | dTSB | SCH TA | dTSBL | 0.2198 | 0.5673 | 1.0000 |
| SCH TA | dTSB | SCH TA | TSB | -5.3507 | 0.5714 | <.0001 |
| SCH TA | dTSBF | SCH TA | dTSBL | 0.3380 | 0.5673 | 1.0000 |
| SCH TA | dTSBF | SCH TA | TSB | -5.2324 | 0.5714 | <.0001 |
| SCH TA | dTSBL | SCH TA | TSB | -5.5704 | 0.5714 | <.0001 |
| SA | dTSB | SA | dTSBF | -0.08525 | 0.5673 | 1.0000 |
| SA | dTSB | SA | dTSBL | -1.3512 | 0.5673 | 1.0000 |
| SA | dTSB | SA | TSB | -1.2124 | 0.5714 | 1.0000 |
| SA | dTSBF | SA | dTSBL | -1.2660 | 0.5673 | 1.0000 |
| SA | dTSBF | SA | TSB | -1.1272 | 0.5714 | 1.0000 |
| SA | dTSBL | SA | TSB | 0.1388 | 0.5714 | 1.0000 |

^a^: Regression coefficient.

^b^: Standard Error.

^c^: Bonferroni-corrected *P*-value.

^d^ *Staphylococcus chromogones* isolate from bulk tank milk

^e^ *Staphylococcus chromogones* isolate from composite cow milk

^f^ *Staphylococcus equorum* isolate from bulk tank milk

^g^ *Staphylococcus equorum* isolate from composite cow milk

^h^ *Staphylococcus chromogenes* isolate causing chronic intramammary infection

^I^ *Staphylococcus chromogenes* isolate from a teat apex of a heifer
