# Supplementary material for: Phenotypic and genotypic assessment of iron acquisition in diverse bovine-associated non-aureus staphylococcal strains
Source: Vet Res. 2024 Jan 12;55:6. doi: 10.1186/s13567-023-01260-z (PMC10785429; doi:10.1186/s13567-023-01260-z)
Supplement: Supplementary file 3 — Additional file 3: Multiple comparisons for the strains from the statistical analysis for strain effect. This includes four field strains: S. chromogenes CCM (SCH CCM), S. chromogenes BTM (SCH BTM), S. equorum CCM (SEQ CCM), and S. equorum BTM (SEQ BTM); two comparative strains: S. chromogenes IM (SCH IM) and S. chromogenes TA (SCH TA); two positive controls: Escherichia coli ATCC 25922 (EC) and Staphylococcus aureus ATCC 25923 (SA). [file 13567_2023_1260_MOESM3_ESM.docx]

|  | **Strain** |  | **Strain** |  | **β^a^** |  | **SE^b^** | **Adj P^c^** |
| --- | --- | --- | --- | --- | --- | --- | --- | --- |
|  | SA |  | SCH CCM |  | 44.7292 |  | 6.0720 | <.0001 |
|  | SA |  | SCH BTM |  | 59.7452 |  | 6.0720 | <.0001 |
|  | SA |  | SEQ CCM |  | 70.8716 |  | 6.0720 | <.0001 |
|  | SA |  | SEQ BTM |  | 71.4149 |  | 6.0720 | <.0001 |
|  | SA |  | SCH IM |  | 52.6683 |  | 6.0720 | <.0001 |
|  | SA |  | SCH TA |  | 72.4298 |  | 6.0720 | <.0001 |
|  | SA |  | EC |  | 12.0171 |  | 6.0720 | 1.0000 |
|  | SCH CCM**^d^** |  | SCH BTM |  | 15.0160 |  | 6.0720 | 0.5844 |
|  | SCH CCM |  | SEQ CCM |  | 26.1424 |  | 6.0720 | 0.0068 |
|  | SCH CCM |  | SEQ BTM |  | 26.6857 |  | 6.0720 | 0.0054 |
|  | SCH CCM |  | SCH IM |  | 7.9392 |  | 6.0720 | 1.0000 |
|  | SCH CCM |  | SCH TA |  | 27.7006 |  | 6.0720 | 0.0035 |
|  | SCH CCM |  | EC |  | -32.7121 |  | 6.0720 | 0.0004 |
|  | SCH BTM**^e^** |  | SEQ CCM |  | 11.1264 |  | 6.0720 | 1.0000 |
|  | SCH BTM |  | SEQ BTM |  | 11.6697 |  | 6.0720 | 1.0000 |
|  | SCH BTM |  | SCH IM |  | -7.0768 |  | 6.0720 | 1.0000 |
|  | SCH BTM |  | SCH TA |  | 12.6846 |  | 6.0720 | 1.0000 |
|  | SCH BTM |  | EC |  | -47.7281 |  | 6.0720 | <.0001 |
|  | SEQ CCM**^f^** |  | SEQ BTM |  | 0.5434 |  | 6.0720 | 1.0000 |
|  | SEQ CCM |  | SCH IM |  | -18.2032 |  | 6.0720 | 0.1746 |
|  | SEQ CCM |  | SCH TA |  | 1.5582 |  | 6.0720 | 1.0000 |
|  | SEQ CCM |  | EC |  | -58.8544 |  | 6.0720 | <.0001 |
|  | SEQ BTM**^g^** |  | SCH IM |  | -18.7466 |  | 6.0720 | 0.1411 |
|  | SEQ BTM |  | SCH TA |  | 1.0149 |  | 6.0720 | 1.0000 |
|  | SEQ BTM |  | EC |  | -59.3978 |  | 6.0720 | <.0001 |
|  | SCH IM**^h^** |  | SCH TA**^i^** |  | 19.7615 |  | 6.0720 | 0.0942 |

^a^ Regression coefficient.

^b^ Standard Error.

^c^ Bonferroni-corrected *P*-value.

^d^ *Staphylococcus chromogones* isolate from composite cow milk

^e^ *Staphylococcus chromogones* isolate from bulk tank milk

^f^ *Staphylococcus equorum* isolate from composite cow milk

^g^ *Staphylococcus equorum* isolate from bulk tank milk

^h^ *Staphylococcus chromogenes* isolate causing chronic intramammary infection

^i^ *Staphylococcus chromogenes* isolate from a teat apex of a heifer
