# Supplementary material for: Phenotypic and genotypic assessment of iron acquisition in diverse bovine-associated non-aureus staphylococcal strains
Source: Vet Res. 2024 Jan 12;55:6. doi: 10.1186/s13567-023-01260-z (PMC10785429; doi:10.1186/s13567-023-01260-z)
Supplement: Supplementary file 4 — Additional file 4: Comparative genomic analysis of the four field strains and two comparative strains, an overview. [file 13567_2023_1260_MOESM4_ESM.docx]

| **Bacterial species** | **Isolate ID** | **Average coverage X** | **Longest contig (Mbps)** | **Genome size (Mbps)** | **G+C content (%)** | **No. of CDS^a^** | **Accession number** |
| --- | --- | --- | --- | --- | --- | --- | --- |
| *S. chromogenes* | IM^b^ | 370 | 2.34 | 2.37 | 36.7 | 2277 | CP133244-CP133246 |
|  | TA^c^ | 263 | 2.37 | 2.39 | 36.8 | 2302 | CP133247-CP133248 |
|  | CCM^d^ | 141 | 2.39 | 2.39 | 36.8 | 2371 | CP133240-CP133241 |
|  | BTM^e^ | 168 | 2.27 | 2.30 | 36.7 | 2194 | CP133242-CP133243 |
| *S. equorum* | CCM^f^ | 719 | 2.65 | 2.73 | 33.0 | 2649 | CP133235-CP133239 |
|  | BTM^g^ | 175 | 2.64 | 2.74 | 33.0 | 2676 | CP133229-CP133234 |

^a^ Number of coding sequences

^b^ *Staphylococcus chromogenes* isolate causing chronic intramammary infection

^c^ *Staphylococcus chromogenes* isolate from a teat apex of a heifer

^d^ *Staphylococcus chromogones* isolate from composite cow milk

^e^ *Staphylococcus chromogones* isolate from bulk tank milk

^f^ *Staphylococcus equorum* isolate from composite cow milk

^g^ *Staphylococcus equorum* isolate from bulk tank milk
